# Shape dependent cytotoxicity of PLGA-PEG nanoparticles on human cells

**DOI:** 10.1038/s41598-017-07588-9

**Published:** 2017-08-04

**Authors:** Bokai Zhang, Ping Sai Lung, Saisai Zhao, Zhiqin Chu, Wojciech Chrzanowski, Quan Li

**Affiliations:** 1 Department of Physics, The Chinese University of Hong Kong, Shatin, New Territory Hong Kong; 2 Pharmacy and Bank Building A15, The University of Sydney, Sydney, Australia

## Abstract

We investigated the influence of nanoparticles’ shape on the physiological responses of cells, when they were fed with spherical and needle-shaped PLGA-PEG nanoparticles (the volume of the nanoparticles had been chosen as the fixed parameter). We found that both types of NPs entered cells via endocytosis and upon internalization they stayed in membrane bounded vesicles. Needle-shaped, but not the spherical-shaped NPs were found to induce significant cytotoxicity in the cell lines tested. Our study evidenced that the cytotoxicity of needle-shaped NPs was induced through the lysosome disruption. Lysosome damage activated the signaling pathways for cell apoptosis, and eventually caused DNA fragmentation and cell death. The present work showed that physiological response of the cells can be very different when the shape of the fed nanoparticles changed from spherical to needle-like. The finding suggests that the toxicity of nanomaterials also depends on their shape.

## Introduction

Cytotoxicity is an important measure in both evaluating the impact of nanomaterial on public health and developing them for various biomedical applications, such as drug delivery and bio-sensing. Many works could be found in the literature trying to establish the correlation between specific material parameters and cell physiological responses/viability, and most of them focused on the surface charge, chemistry and size of the NPs. The cytotoxicity induced by NP surface charge was a result of Columbic interaction, i.e., the negatively charged plasma membrane attracted to positively charged NPs, which could cause membrane disruption and/or proton pump effect^1, 2^. For example, positively charged gold NPs depolarized cell membrane to the greatest extent while NPs of other charges had negligible effect^3^. Surface chemistry induced cytotoxicity had many different origins, including de-activation of biomolecules due to specific surface binding, non-specific protein binding and their denaturation (i.e. beta-sheet formation)^4^, membrane perturbation induced temperature/pH changes, and direct release of various toxin^5^. For instance, magnetic iron NPs coated with dendritic guanidines resulted in a similar cell penetration ability as human immunodeficiency virus-1 transactivator (HIV-TAT) peptide^6^. Reports on size induced cytotoxicity were more complicated, as more than one material parameter was usually involved. Nonetheless, some reports suggested that the size effect is directly linked to the chemistry due to the different surface activity (chemical) related to the specific surface of small particles, as compared to their larger sized counterparts^2, 7^.

While there was strong clinical evidence that shape of NPs had a significant impact on cellular fate (e.g. asbestosis)^8^, the effect of shape of the nanoparticles on cell responses was much less investigated. Relevant work included the toxicity study of carbon nanotubes which were found to induce significant cytotoxicity and even claimed to be ‘new asbestos’^9^. Direct plasma membrane penetration, endosomal leakage, and nuclear translocation had been detected when CNTs were fed to various cell lines^10^. However, differences in the aspect ratio^11, 12^, complicate surface chemistry and charge^9–12^ of the examined CNT samples made it difficult to determine conclusively the origin of the cytotoxicity. Therefore, question whether NPs shape, chemistry, charge, or a specific combination of all possible characteristic contributes to cytotoxicity remained open.

A few polymeric material systems had also been studied in this regard. For example, needle-shape polystyrene particles with dimension of 4.4 × 0.45 µm, ‘blunt’ end, were found to cause transient cell membrane disruption, although cell recovery was identified after 48 h^13^. BSA coated PLGA microneedles were found to enhance green fluorescent protein (GFP) knockdown of GFP expressing endothelial cells after co-incubation with siRNA, which phenomenon was much less significant when PLGA microspheres were employed^14^. Nonetheless, the nature of the shape effect was not fully understood.

In the present work, we investigated the effect of shape of Poly (lactic-co-glycolic acid) polyethylene glycol nanoparticles (PLGA-PEG NPs) on the physiological response of human cells. PLGA is a FDA approved material for biomedical application due to its biodegradability^15, 16^ and biocompatibility^17^. It is a very attractive candidate in drug delivery with features of controlled^18^ and sustained^19^ release, stealth^20^ and targeting^21^. Here we engineered the PLGA-PEG NPs into spherical- or needle-shaped morphologies. Needle-shaped NPs were formed by direct stretching of the as-synthesized spherical NPs in order to maintain the same volume, chemistry and charge. When introduced to cells, the needle-shaped NPs were found to induce a series of physiological changes in cells, which eventually led to significant cytotoxicity. The nature of the shape effect and its induced cytotoxicity pathway were discussed. The present work show that physiological response of the cells can be very different when the shape of the fed nanoparticles changed from spherical to needle-like. The finding suggests that, in addition to the known material parameters such as composition, surface chemistry, and surface charge, shape is also an important parameter affecting the toxicity of nanomaterials.

## Results

### Spherical- and needle-shaped PLGA-PEG nanoparticles

**Figure 1:**
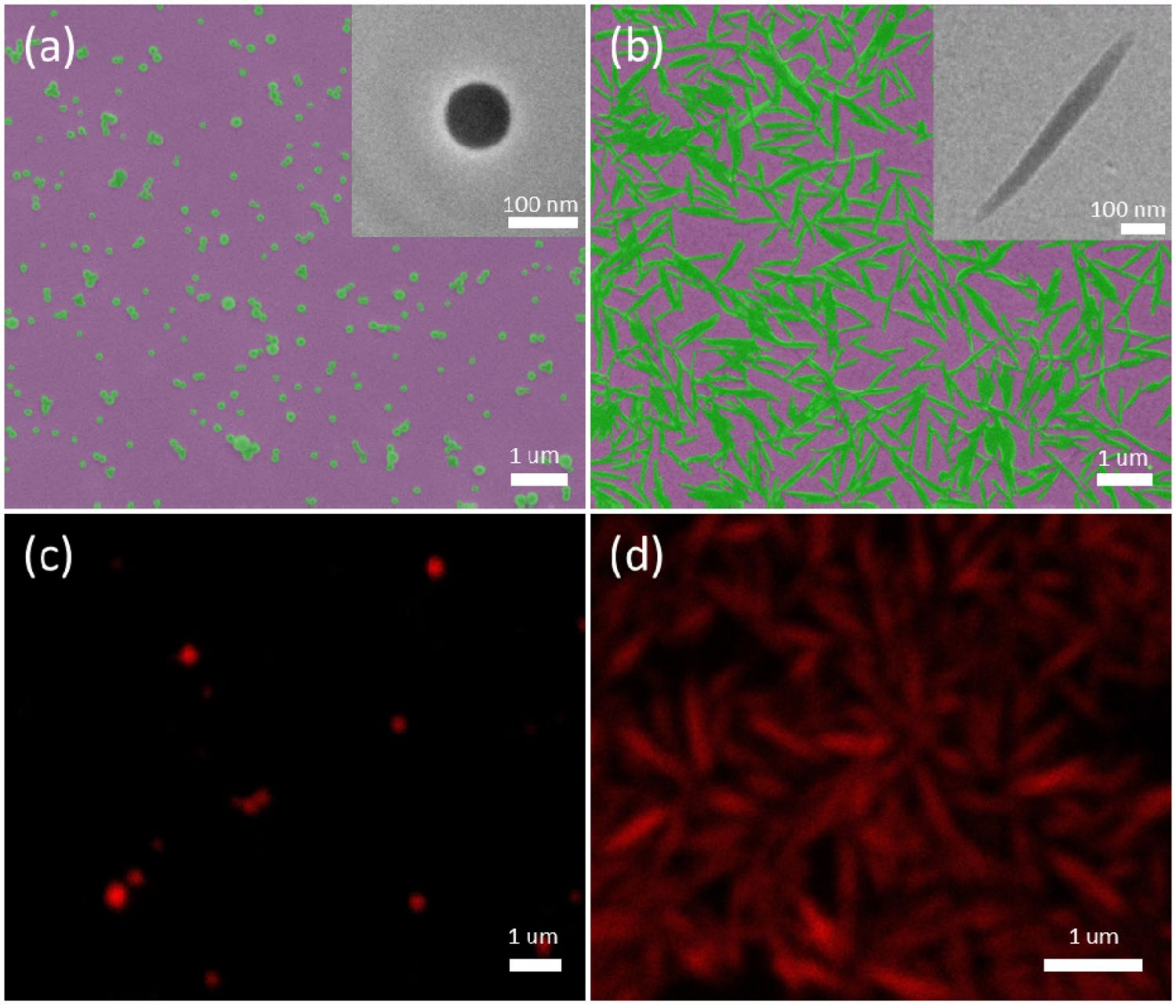
PLGA-PEG NPs with different shapes. SEM images of (**a**) spherical-shaped and (**b**) needle-shaped PLGA-PEG NPs, insert are the magnified TEM images; (**c,d**) Corresponding confocal images showing the fluorescence coming from incorporated Nile Red dye.

Scanning electron microscopy (SEM) images showed that the developed PLGA-PEG NPs have two distinct morphologies: nanospheres (Fig. 1a) and nanoneedles (Fig. 1b). High magnification Transmission electron microscopy (TEM) images further confirmed that desired morphologies were obtained (inset in Fig. 1a and b). The average diameter of the spherical-shaped NPs was 90 nm, while the needle-shaped NPs was 30 nm in diameter and their average aspect ratio was ~18. The apex size of the needle-shaped NPs could be described using their radius of curvature, which was measured by TEM (inset of Fig. 1b), and estimated as ~10 nm. To enable tracking of the NPs during and post internalization into cells using confocal microscopy, Nile Red Dye was incorporated into NPs. The uniform red fluorescence of the NPs was confirmed for both samples (Fig. 1c and d).

**Figure 2:**
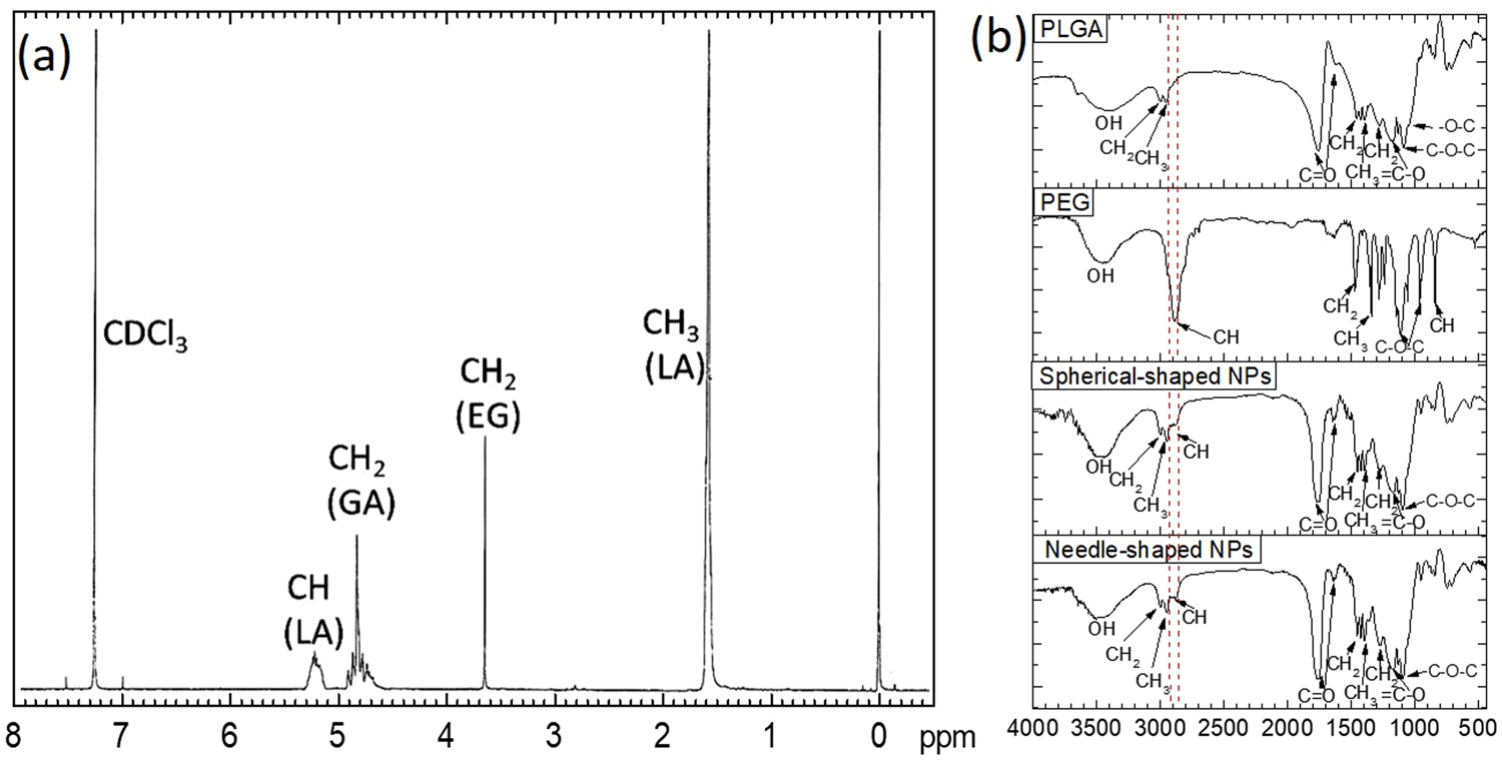
Chemical characterization of PLGA-PEG NPs. (**a**) 1H-NMR spectrum of PLGA-PEG copolymer showing their chemical composition, (**b**) Typical FTIR spectrum of PLGA, PEG, and PLGA-PEG NPs (both spherical and needle-shaped).

The chemical composition of the PLGA-PEG copolymer was evaluated by ^1^H-NMR (Fig. 2a), which showed the characteristic chemical peak values for the -CH_3_, -CH_2_ and -CH groups. The methyl group (-CH_3_), with a peak at 1.66 ppm, was assigned as a reference for the presence of lactic acid (LA) monomer. Methylene group (-CH_2_), with a peak at 4.85 ppm, served as the reference for glycolic acid (GA) monomer. The presence of methyl group (-CH_3_) and methylene group (-CH_2_) indicated the content of PLGA. In addition, the methane group (-CH), with a peak at 3.64 ppm, was the reference for the ethylene glycol (EG) monomer, which suggested the successful poly ethylene glycol (PEG) conjugation to PLGA.

Surface chemistry of spherical- and needle-shaped PLGA-PEG NPs was studied by FTIR (Fig. 2b). In the spectrum of pure PLGA polymer, strong band at around 1750 cm^−1^ corresponded to the stretch of the carbonyl groups (C=O) in the PLGA chain. Bands between 1300 and 1150 cm^−1^ were the asymmetrical and symmetrical vibrations of the C-C(=O)-O in the polymer chain. Band at 3000 cm^−1^ was due to the stretching of –CH_3_ group of LA, band at 2956 cm^−1^ was due to the stretching of –CH_2_ group of GA. In the spectrum of pure PEG, band at 2885 cm^−1^ was originated from –CH stretching of the methylene group in PEG. The in plane C-H deformation from 1185 to 1090 cm^−1^ can also be observed. The spectra taken from both spherical- and needle-shaped PLGA-PEG NPs showed similar features. In particular, the –CH stretching band at 2885 cm^−1^ confirmed the incorporation of PEG in both types of NPs.

Zeta potential measurements of the PLGA-PEG NPs showed that both spherical and needle-shaped NPs were negatively charged, and the measured average zeta potential was almost the same for both shapes (ζ = ~−23 mV, Table S1). This result confirmed that shape engineering of PLGA-PEG NPs did not induce obvious influence on surface charge.

### Cellular uptake and intracellular distribution of PLGA-PEG nanoparticles

**Figure 3:**
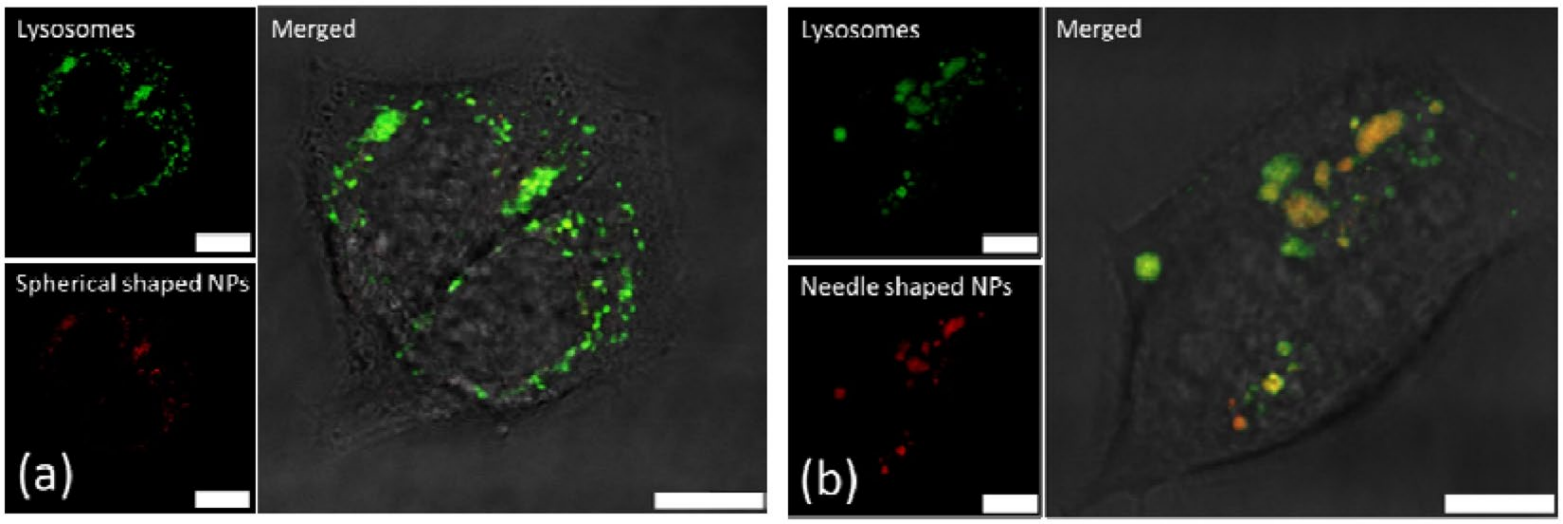
Shape dependent PLGA-PEG NPs intracellular distribution. Confocal images of cellular uptake of (**a**) spherical- and (**b**) needle-shaped NPs at 50 µg/mL by HepG2 cells for 24 hours. (Green: Lysosomes, Red: NPs, Yellow: overlaid. Scale bar: 10 µm).

Human liver carcinoma cells (HepG2) were fed with spherical and needle-shaped NPs at 50 µg/mL for 24 hours. NPs were internalized by the cells, as evidenced by the fluorescence signal (red color) inside the cells (Fig. 3). Most of the florescence signals that originated from NPs overlapped with green fluorescence from lysotracker, suggesting a good overlap between the NPs and the lysosomes. This served as a direct evidence of endocytosis. The cellular uptake amount increased with the feeding concentration for both spherical- and needle-shaped NPs. Nevertheless, more spherical-shaped NPs (more than twice) than their needle-shaped counterpart were engulfed by cells (Figure S1).

### Shape dependent physiological change and cytotoxicity of PLGA-PEG nanoparticles in human cells

**Figure 4:**
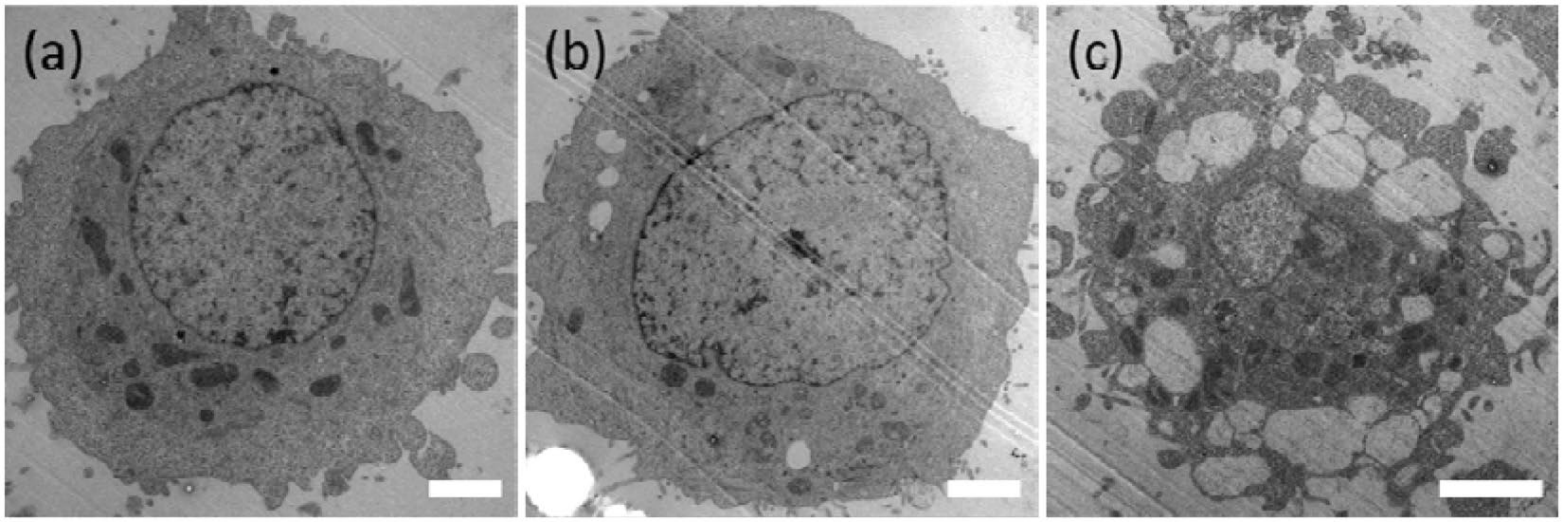
The effects of PLGA-PEG NPs’ shape on cell morphology. TEM images of the morphologies of HepG2 cells after their being fed with (**a**) none (control sample), (**b**) spherical-, and (**c**) needle-shaped NPs at 250 µg/mL for 24 hours. (Scale bar: 2 µm).

Different morphological evolution of the cells was identified after their being incubated with spherical- or needle-shaped NPs at all feeding concentrations (i.e., 25 to 250 µg/ml), and such difference became more significant at higher feeding concentrations. Figure 4 showed typical TEM images taken from cells fed with none (control sample), spherical- and needle-shaped NPs at 250 µg/mL for 24 hours. Enlarged lysosomes were constantly found in cells fed with needle-shaped NPs (Figure S2b), but not in control cells (Fig. 4a) and cells fed with spherical-shaped NPs (Figure S2a), being consistent with the observation made using confocal microscope (Figure S3, S4 & S5). Not only many lysosomes in the needle-shaped NPs fed cells had extremely large size, but also cell blebbing became obvious, causing the ill cell morphology. This was completely absent in the cells fed with spherical-shaped NPs or in the control sample. Similar observation was made by confocal images when using HeLa cells (Figure S6, S7 & S8).

**Figure 5:**
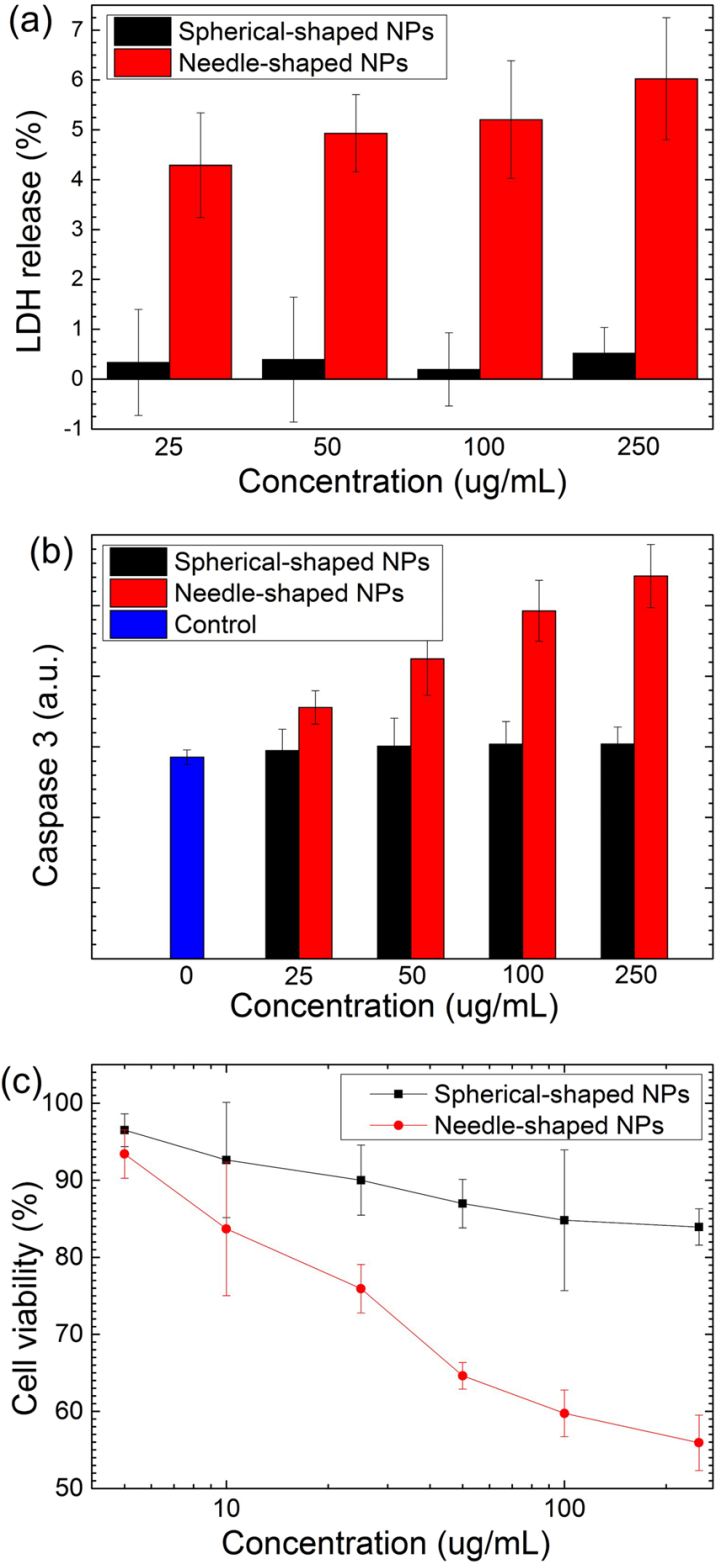
Shape dependent PLGA-PEG NPs’ cytotoxicity. (**a**) LDH release (normalized to lysed control cell), (**b**) Caspase 3 activity and (**c**) MTT assay after HepG2 cells were fed with spherical- and needle-shaped PLGA-PEG NPs.

The abnormally enlarged lysosome suggested possible membrane disruption in cells fed with needle-shaped NPs. We therefore examined the LDH release in the respective cell samples, as LDH served as common indicator for membrane damage^22^. Figure 5a showed the LDH release percentage (normalized to that in the lysed control sample) taken from the cells fed with spherical- or needle-shaped NPs at concentration ranging from 25 to 250 µg/mL for 24 hours. Little LDH release was found in cells fed with spherical-shaped NPs, while ~4 to 6% LDH release was detected in cells fed with needle-shaped NPs, and it increased with the NP feeding concentration. Similar observation was made when using HeLa cells (Figure S9a).

A possible consequence of lysosome membrane disruption is the activation of cleaved caspase 3^23, 24^. To check this, we quantitatively studied the cytosol expression of cleaved caspase 3 in the corresponding cells. Figure 5b showed the cleaved caspase 3 activity of the control cells and cells fed with spherical- and needle-shaped NPs at concentration ranging from 25 to 250 µg/mL for 24 hours. Spherical-shaped NPs fed cells had caspase 3 activity similar to that of the control cells, while more significant caspase 3 activity was observed in cells fed with needle-shaped NPs, and such activity increased with the feeding concentration. Similar observation was made when using HeLa cells (Figure S9b).

The cytotoxicity was evaluated by MTT assay. Figure 5c gave the MTT results taken from cells fed with spherical- and needle-shaped NPs at concentration ranging from 5 to 250 µg/mL for 24 hours. While little cytotoxicity was observed in cells fed with spherical-shaped NPs (cell viability remained at around 84% at high feeding concentration (250 µg/mL)), cytotoxicity that increased with the NPs feeding concentration was identified in cells fed with needle-shaped NPs, and it became very significant at high feeding concentrations (cell viability decreased from around 93% at concentration of 5 µg/mL to around 56% at concentration of 250 µg/mL). Similar observation was made when using HeLa cells (Figure S9c).

**Figure 6:**
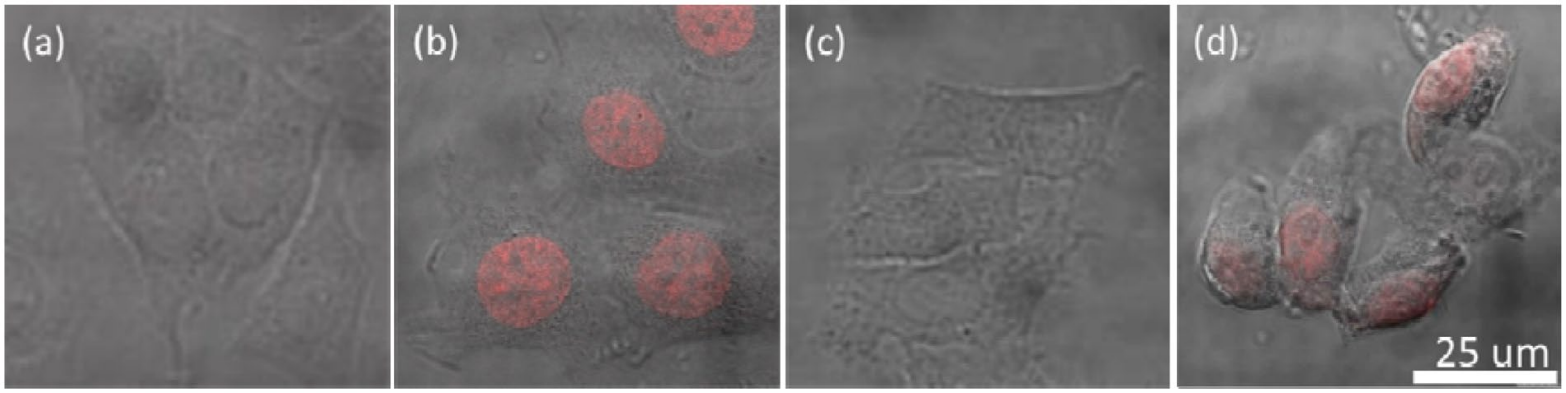
Needle-shaped PLGA-PEG NPs induced DNA fragmentation by TUNEL assay. Confocal images (merged with transmitted channel) of HepG2 cells (stained with TUNEL Red) after their being fed with (**a**) & (**b**) none, (negative and positive control), (**c**) spherical- and (**d**) needle-shaped NPs at 250 µg/mL for 24 hours. (Red: TUNEL. All the figures share the same scale bar).

As a common method to detect DNA fragmentation resulting from apoptotic signaling cascades, TUNEL assay was employed to further investigate the cytotoxicity and the possible DNA damage induced by PLGA-PEG NPs. DNA fragmentation was observed in cells fed with needle-shaped NPs at all concentration range tested (Figure S11), and was the most obvious at high feeding concentration (250 µg/ml, Fig. 6), while that no DNA fragmentation was detected in cells fed with spherical-shaped NPs even at high feeding concentration (250 µg/mL, Fig. 6 & S10).

## Discussion

Although both spherical- and needle-shaped PLGA-PEG NPs entered cells via endocytosis, the former one showed higher cellular uptake amount. Similar shape dependent cellular uptake had been reported in the literature, and was explained by the different membrane bending energies required for entry of NPs with different shapes^25^. Then it is interesting to note that even with lower cellular uptake amount, the needle-shaped NPs induced much more significant cytotoxicity than its spherical counterpart. Obvious physiology changes of the cells started from the lysosomal membrane disruption, as evidenced in enlarged lysosomes, detectable LDH release, and the enhanced activation of cleaved caspase 3, when the needle-shaped NPs were fed to them.

It is important to note that the spherical- and needle-shaped NPs shared similar volume, surface chemistry and charge, as the needle-shaped ones were obtained by stretching the spherical-shaped ones. The only difference between the two types of NPs was their shape. In particular, sharp ends presented in the needle-shaped, but not the spherical-shaped NPs. It had been suggested that local sharpness of the NPs significantly affected their interaction with the lipid bilayer membrane—energy penalty determined that nanodiamond with small radius of curvature at the end would “sink” into the plasma membrane, while the ones with large radius of curvature stayed above the membrane^26^. This hypothesis provided a possible origin for the observed lysosome membrane disruption in the case of needle-shaped NPs.

In fact, experimental evidence showed that nanodiamond indeed cut through the vesicle membrane and were released to cytoplasm, shortly after their cellular entry^27^. Nevertheless, such membrane rupture did not cause much cytotoxicity in the case of nanodiamond, mainly due to the fact that NDs’ cytosol escape took place at the early endosome stage, which usually caused little damage to the cell^27^. As a comparison, the local sharpness of needle-shaped PLGA-PEG NPs failed to rupture the vesicle membrane inside the cells. This might be due to the significantly different stiffness of the PLGA-PEG (in the range of 2–80 MPa^28, 29^) from that of diamond (130 GPa^30^). Mechanisms of how stiffness of PLGA-PEG NPs affected their interaction with the vesicle membrane remain unclear and required further investigation.

**Figure 7:**
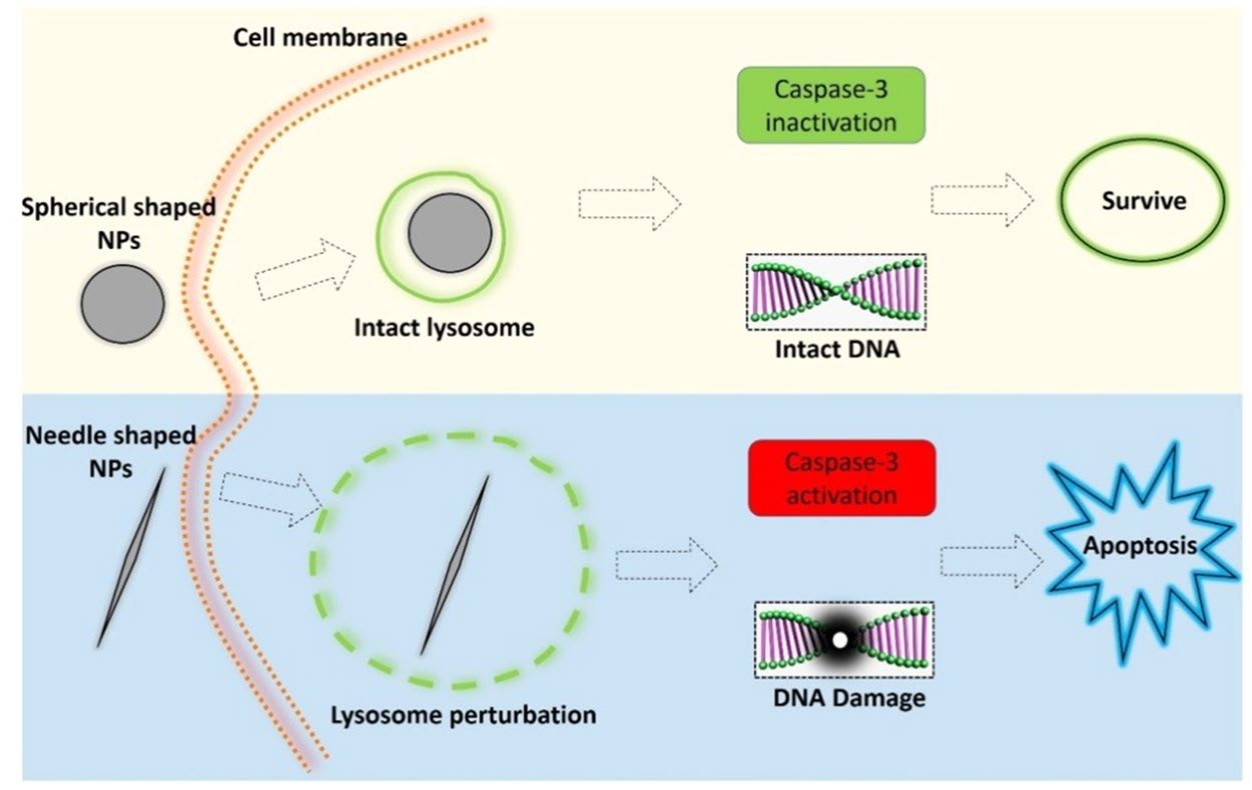
Schematic representation of the proposed cytotoxicity pathways for PLGA-PEG NPs with different morphological features. Upper panel: A spherical-shaped PLGA NP entered the cell via endocytosis, stably resided in the lysosomes, and had no apparent cytotoxicity. Lower panel: A needle-shaped PLGA NP entered the cell via endocytosis, resided in lysosomes and induced lysosomal membrane perturbation, caused caspase-3 activation and DNA damage, and finally apoptosis.

On the other hand, although the vesicle membrane was not ruptured, perturbation to the lysosomes persisted as indicated by their observed enlargement (Figs 3 & 4). It had been found that the lysosomal membrane perturbation would cause the release of cathepsins and other hydrolases from the lysosomal lumen to the cytosol, resulting in digestion of vital proteins, initializing DNA fragmentation and apoptotic signaling^31, 32^. The experimental results suggested the following cytotoxicity pathway (Fig. 7) as induced by the needle-shaped PLGA-PEG NPs: local sharpness of the NPs induced the lysosomal membrane disruption, followed by the activation of caspase 3 and finally resulted in apoptosis and DNA damage.

## Conclusion

We fabricated both spherical- and needle-shaped PLGA-PEG NPs, and investigated the impact of their shapes on the physiological response of the cells. Experimental evidence showed that, only the needle-shaped PLGA-PEG NPs induced lysosomal membrane disruption, caused lysosome enlargement, and subsequently the activation of caspase-3 and DNA damage, both of which eventually led to cell apoptosis. The current study alerted the importance of NPs‘ shape effect, which was underestimated in many cases.

## Methods

### Synthesis and characterization of PLGA-PEG NPs

Synthesis of PLGA-PEG NPs were started from the PEGylation of PLGA polymer, followed with nano-precipitation/solvent diffusion method^33^ to synthesize spherical-shaped NPs. The needle-shaped NPs were stretched from the as-synthesized spherical-shaped NPs via the stretching method^34^. For details refer to SI.

The conjugation of PEG to PLGA was verified by 1H-Nuclear Magnetic Resonance. The morphology and size of the PLGA-PEG NPs were investigated by SEM (FEI Quanta 400 FEI microscope). Surface chemistry of the PLGA-PEG NPs was studied by FTIR. Surface charge of the PLGA-PEG NPs was evaluated in PBS by DLS. To verify the conjugation of Nile Red to PLGA-PEG NPs, the photoluminescence of aqueous solution containing NPs was measured with photoluminescence (Hitachi P7000).

### Introducing PLGA NPs to human cells

HepG2 cells were used in this study. The cells were cultured with Dulbecco’s modified Eagle’s media (Life technology, HK) and 10% fetal bovine serum (Life technology, HK). All PLGA-PEG NPs were sterilized before using by UV light for 15 min. Cells were seeded and incubated for 24 hours before the NPs were introduced. The feeding concentration of the NPs was 25, 50, 100 and 250 µg/mL unless otherwise specified.

### Characterization of PLGA NPs interacting with human cells

For confocal microscopy study, the NPs fed cells were washed with PBS twice to eliminate free NPs that were not taken up by cells. 2 mL DMEM containing 0.005% vol/vol Lysotracker green (Invitrogen) was added to the dish, and 1 hour of incubation was allowed, then the cells were examined with confocal microscope (Leica SP5TCS II) with a 63 × water-immersion objective lens.

For transmission electron microscopy study, the NP-fed cells were fixed using typical procedures^35^ at the end of their incubation with NPs. Then, cells were dehydrated in a grade series of ethanol and embedded in Spurr resin. The resin blocks were sectioned by using an ultramicrotome (Leica) and the 90 nm thickness sections were transferred onto TEM grid (TED Pella Inc. USA). The grids were further stained with uranyl acetate and lead acetate solution. The cell samples were observed using TEM (FEI TS12).

All biochemical assays were started from seeding the cells in 96 well plate for 24 hours, then adding the NPs for another 24 hours of incubation. Cell membrane perturbation was studied by LDH release assay (CytoTox 96 Non-Radioactive Cytotoxicity Assay, Promega), the apoptotic toxicity was investigated by Caspase 3 activity assay (Caspase-Glo 3/7 Assay, Promega), the cytotoxicity of the cells was evaluated using MTT assay.

NDA fragmentation was studied by using Terminal transferase deoxy-UTP Nick End Labeling (TUNEL, *In Situ* Cell death Detection Kit-TMR red assay kit (#12156792910, Roche, USA)).

## Electronic supplementary material

Supplementary information
