# Supplementary material for: Shape dependent cytotoxicity of PLGA-PEG nanoparticles on human cells
Source: Sci Rep. 2017 Aug 4;7:7315. doi: 10.1038/s41598-017-07588-9 (PMC5544670; doi:10.1038/s41598-017-07588-9)
Supplement: Supplementary file 1 — Supplementary information [file 41598_2017_7588_MOESM1_ESM.pdf]

Supporting Information

Shape dependent cytotoxicity of PLGA-PEG nanoparticles on human cells

Bokai Zhang[1], Ping Sai Lung[1], Saisai Zhao[1], Zhiqin Chu[1], Wojciech Chrzanowski[2], Quan Li[1]*.

[1]Department of Physics, The Chinese University of Hong Kong, Shatin, New Territory, Hong Kong.

[2]Pharmacy and Bank Building A15, The University of Sydney, Sydney, Australia.

*corresponding. liquan@phy.cuhk.edu.hk

Synthesis of PLGA-PEG NPs

The PEGylation of PLGA was carried out by the conjugation of PLGA (PLGA acid end/PLGA-COOH, Sigma-Aldrich) to PEG (NH_2-PEG5000-SH, JenKem Technology, Shahai)[1]. 200 mg PLGA was dissolved in 0.8 mL DCM for at least 3 hours. 9.2 mg EDC was added to this polymer solution under gentle stirring for 15 min, followed by the addition of 5.4 mg NHS under gentle stirring for 16 hours. The preactivated PLGA-NHS was precipitated with ice-cold diethyl ether and centrifuged at 4000 rpm for 5 min at 4 °C. The residue was washed three times with an ice-cold 1:1 mixture of diethyl ether and methanol. The washed precipitation (PLGA-NSH) was dried under vacuum and stored at -20 °C for later use. To synthesize PLGA-PEG, 150 mg PLGA-NSH was dissolved in 0.8 mL DCM, 50 mg PEG (NH_2-PEG5000-SH) was added after total dissolving PLGA-NSH. The solution was stirred for 24 hours. The synthesized PLGA-PEG was precipitated with ice-cold methanol and centrifuged at 4000 rpm for 5 min at 4 °C. Followed by 3 times wash with ice-cold methanol to remove the unreacted PEG. At last, the polymer was dried under vacuum and stored at -20 °C for further use.

Spherical-shaped PLGA-PEG NPs loaded with the Nile Red (Invitrogen) were synthesized via the nano-precipitation/solvent diffusion method[2] with slight modification. Briefly, 100 mg PLGA-PEG and 1 mg Nile Red were dissolved in 2.5 mL of a 3:2 mixture of DCM and acetone. This polymer solution was added to 10 mL of 5 % PVA solution dropwisely under magnetic stirring at 9000 rpm, then, the solution was homogenized for 1 hour using a sonciator bath to generate an oil-in-water (O/W) emulsion. After that, the emulsion was added to 50 mL of DI water under gentle stirring for 4 hours at room temperature to evaporate the organic solvent. After removing the remaining solvents, the NPs were collect by centrifugation at 15000 g force and passed through 0.45 um filter, resulting in 90 nm diameter spherical-shaped PLGA-PEG NPs. The NPs were freeze dried and stored at 4 °C for later use.

Needle-shaped PLGA-PEG NPs are synthesized via the stretching method[3] with slight modification. Generally, 1 % glycerol was added in 10 % PVA solution. The spherical-shaped PLGA-PEG NPs were added to this mixture to a concentration up to 0.1 % wt/vol. 15 mL of the solution was dried on a 12 × 16 cm^2 flat surface to form an 80 µm thickness film. The film was cut into sections, and then stretched in one direction at the temperature higher than 70 °C by using a custom-made apparatus. The stretched films were dissolved in DI water. The particles were washed by centrifugation at 12000 g force with the same solution for at least 5 times to remove all PVA from the surface of the particles. The particles were finally freeze dried, weighted and stored at -20 °C for further use.

	Spherical-shaped NPs	Needle-shaped NPs
Zeta potential (mV)	-22.46±1.53	-24.09±0.76

Table S1. Zeta potential of spherical- and needle-shaped PLGA-PEG NPs.

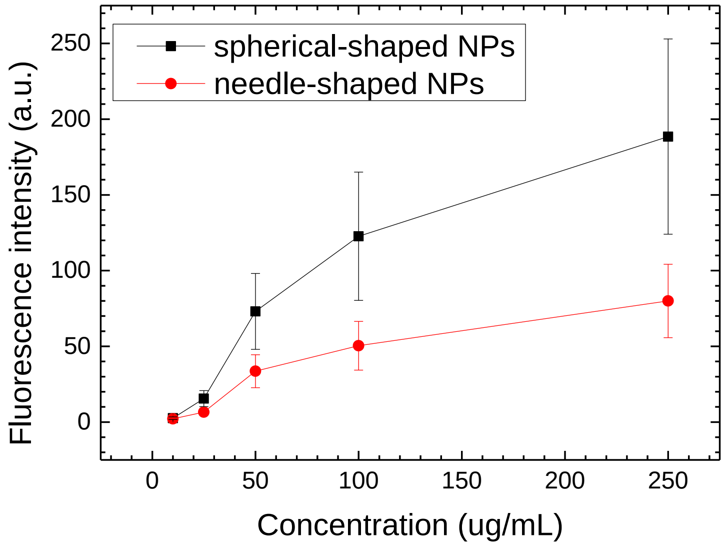

Figure S1. Concentration dependent cellular uptake of spherical- and needle-shaped NPs by HepG2 cells for 24 hours measured by flow cytometry.

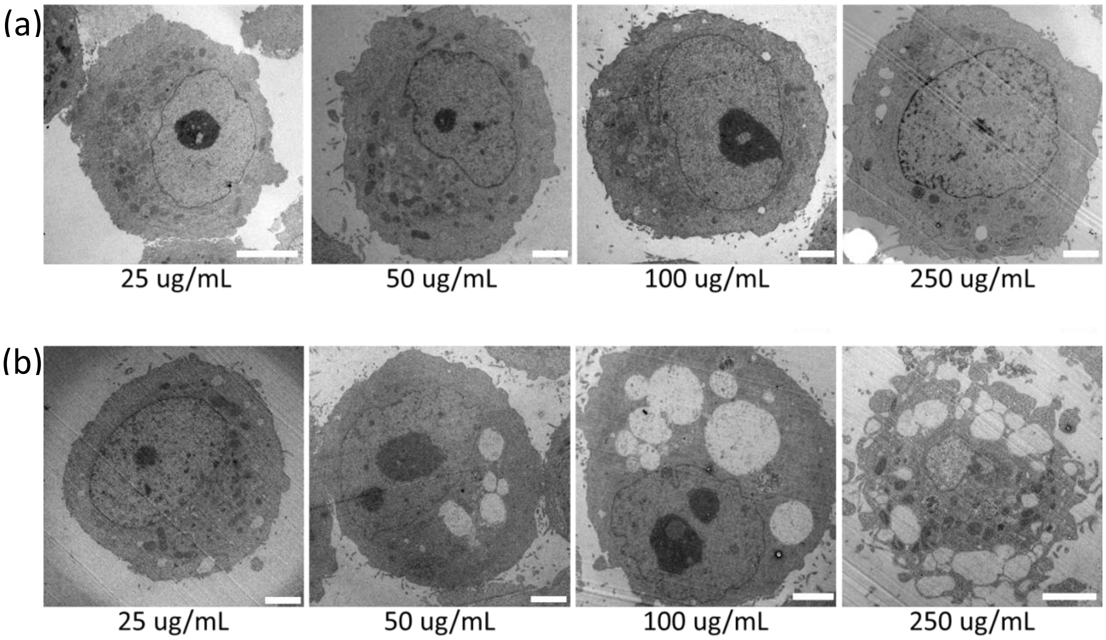

Figure S2. TEM images showing the morphological change of HepG2 cells fed with (a) spherical- and (b) needle-shaped NPs at different feeding concentration for 24 hours, the percentage of cells showing the blebbing morphology (fed with 250 μg/mL needle-shaped NPs) was ~10% in 40 cells (TEM sample) examined. (Scale bar: 2 μm)

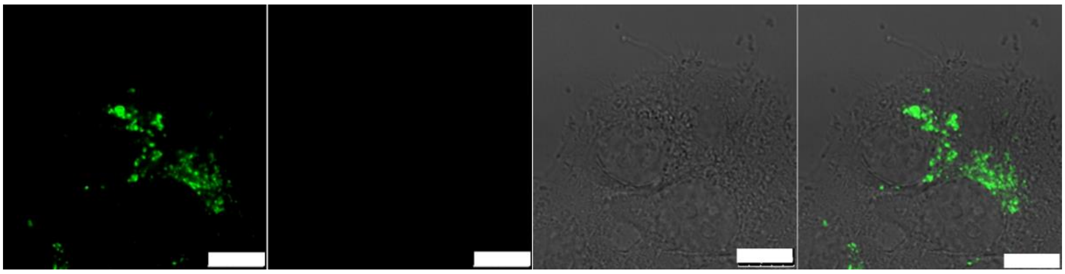

Figure S3. Confocal images of HepG2 cells (no nanoparticle feeding, control sample) for 24 hours (Images from left to right: lysosome in green, NPs in red, transmission and overlaid. Scale bar: 10 μm).

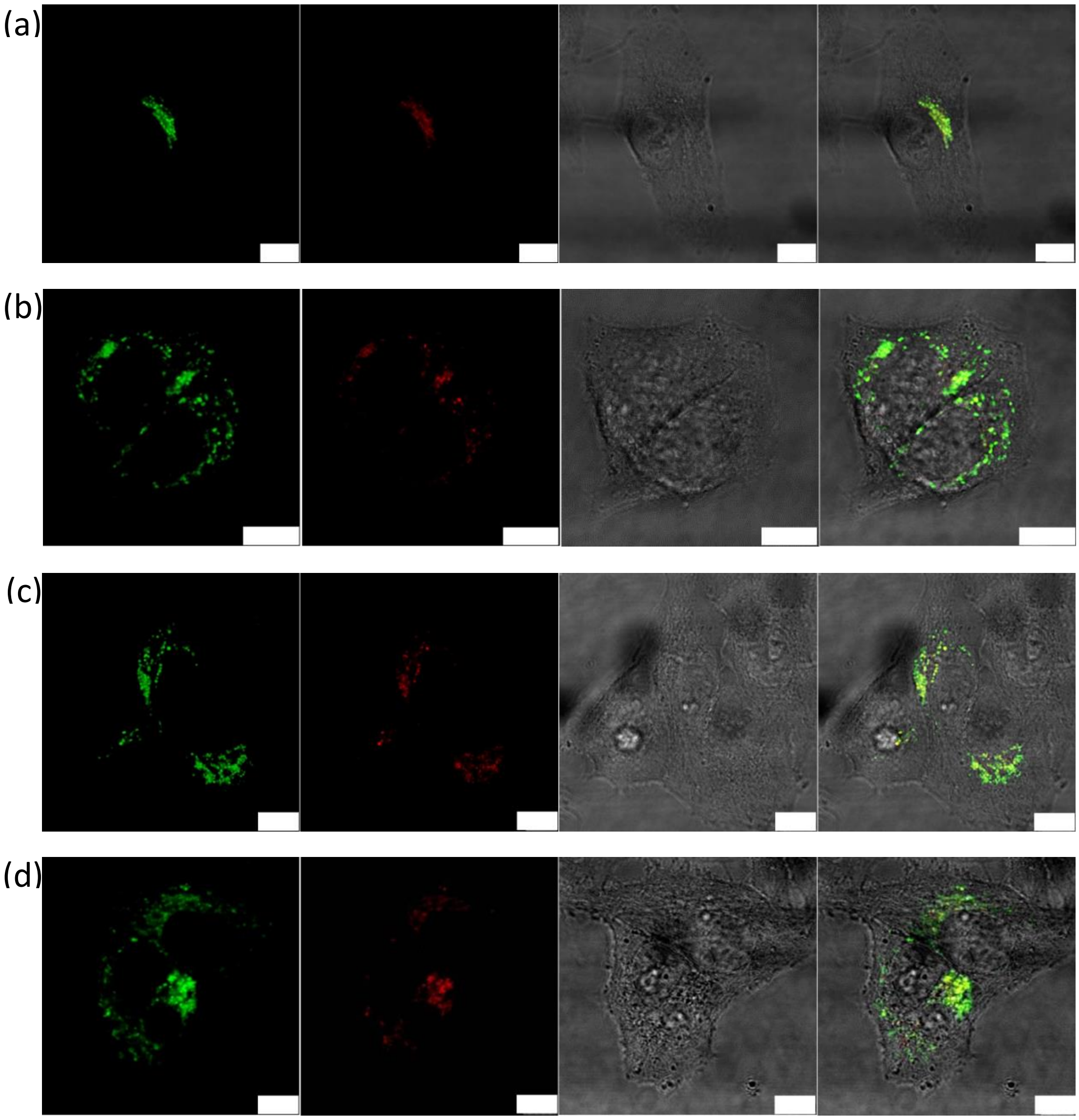

Figure S4. Confocal images of HepG2 cells fed with spherical-shaped NPs at different concentration for 24 hours. (a) 25, (b) 50, (c) 100, (d) 250 μg/mL. (Images from left to right: lysosome in green, NPs in red, transmission and overlaid. Scale bar: 10 μm).

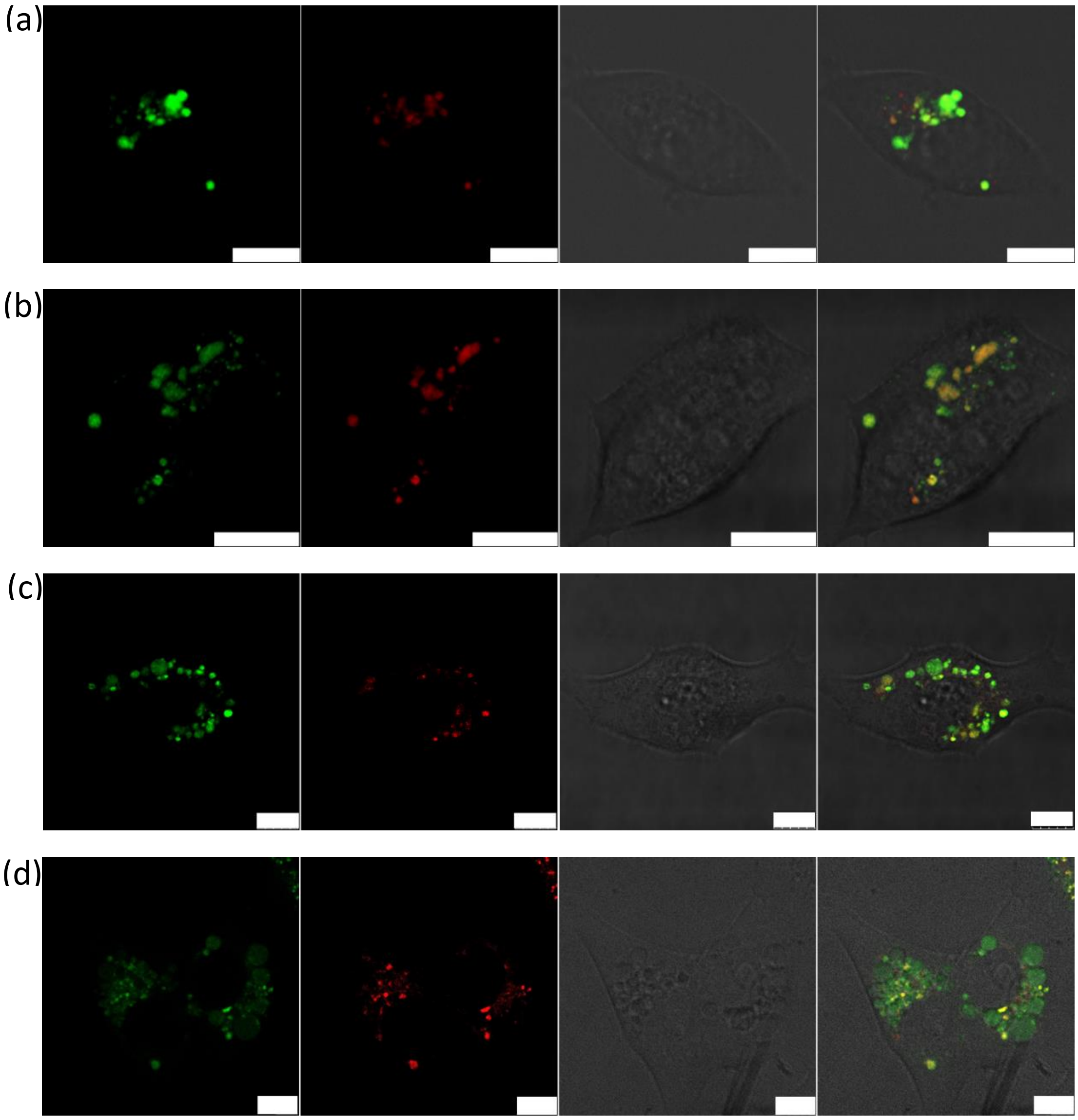

Figure S5. Confocal images of HepG2 cells fed with needle-shaped NPs at different concentration for 24 hours. (a) 25, (b) 50, (c) 100, (d) 250 μg/mL. (Images from left to right: lysosome in green, NPs in red, transmission and overlaid. Scale bar: 10 μm).

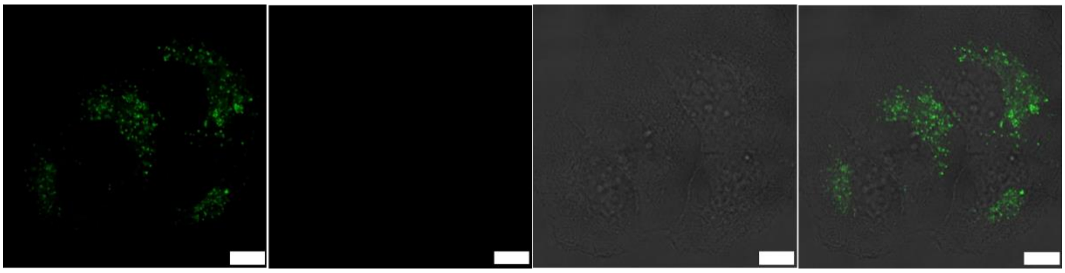

Figure S6. Confocal images of HeLa cells (no NP feeding, control sample) for 24 hours (Images from left to right: lysosome in green, NPs in red, transmission and overlaid. Scale bar: 10 μm).

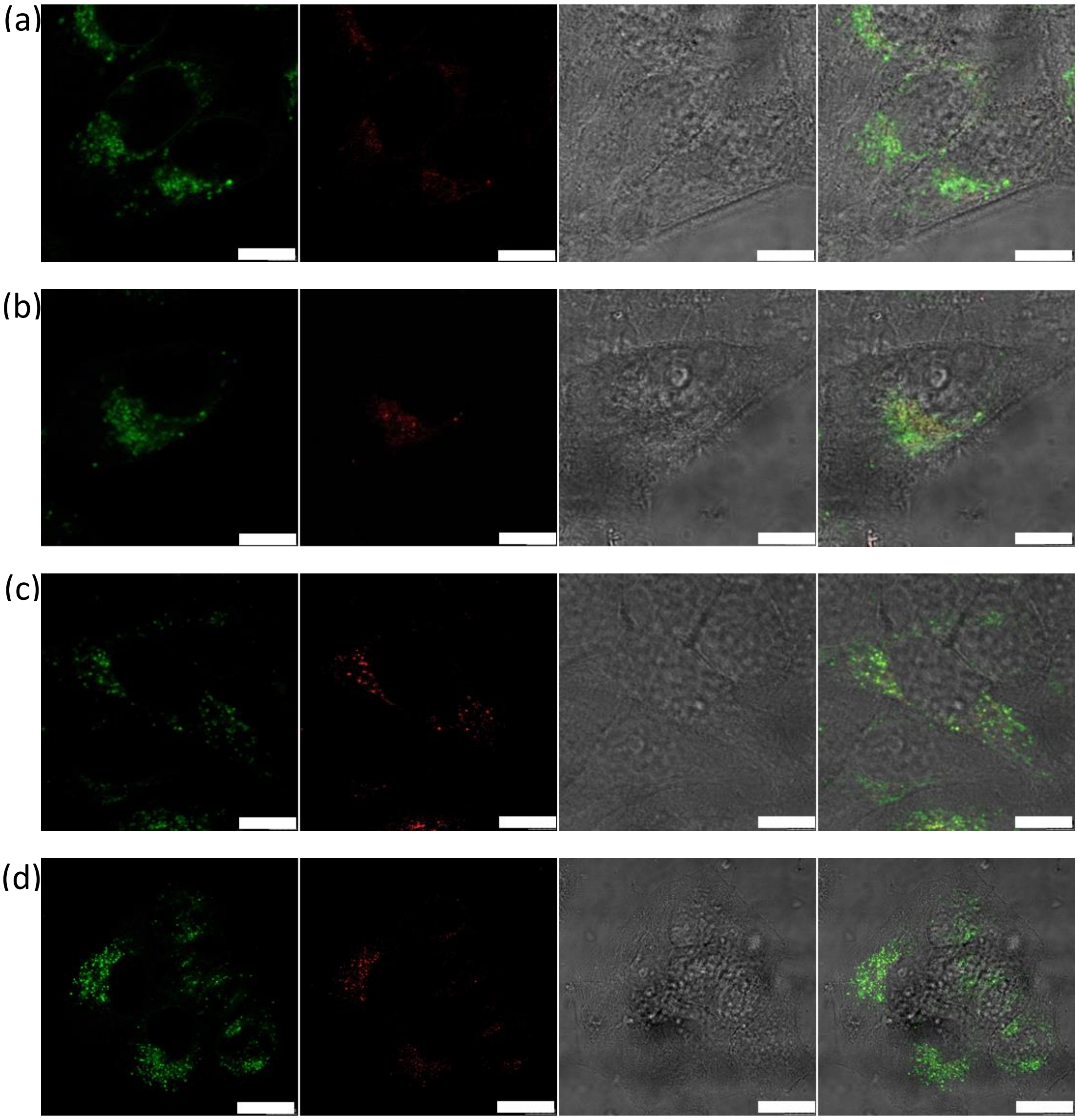

Figure S7. Confocal images of HeLa cells fed with spherical-shaped NPs at different concentration for 24 hours. (a) 25, (b) 50, (c) 100, (d) 250 μg/mL. (Images from left to right: lysosome in green, NPs in red, transmission and overlaid. Scale bar: 10 μm).

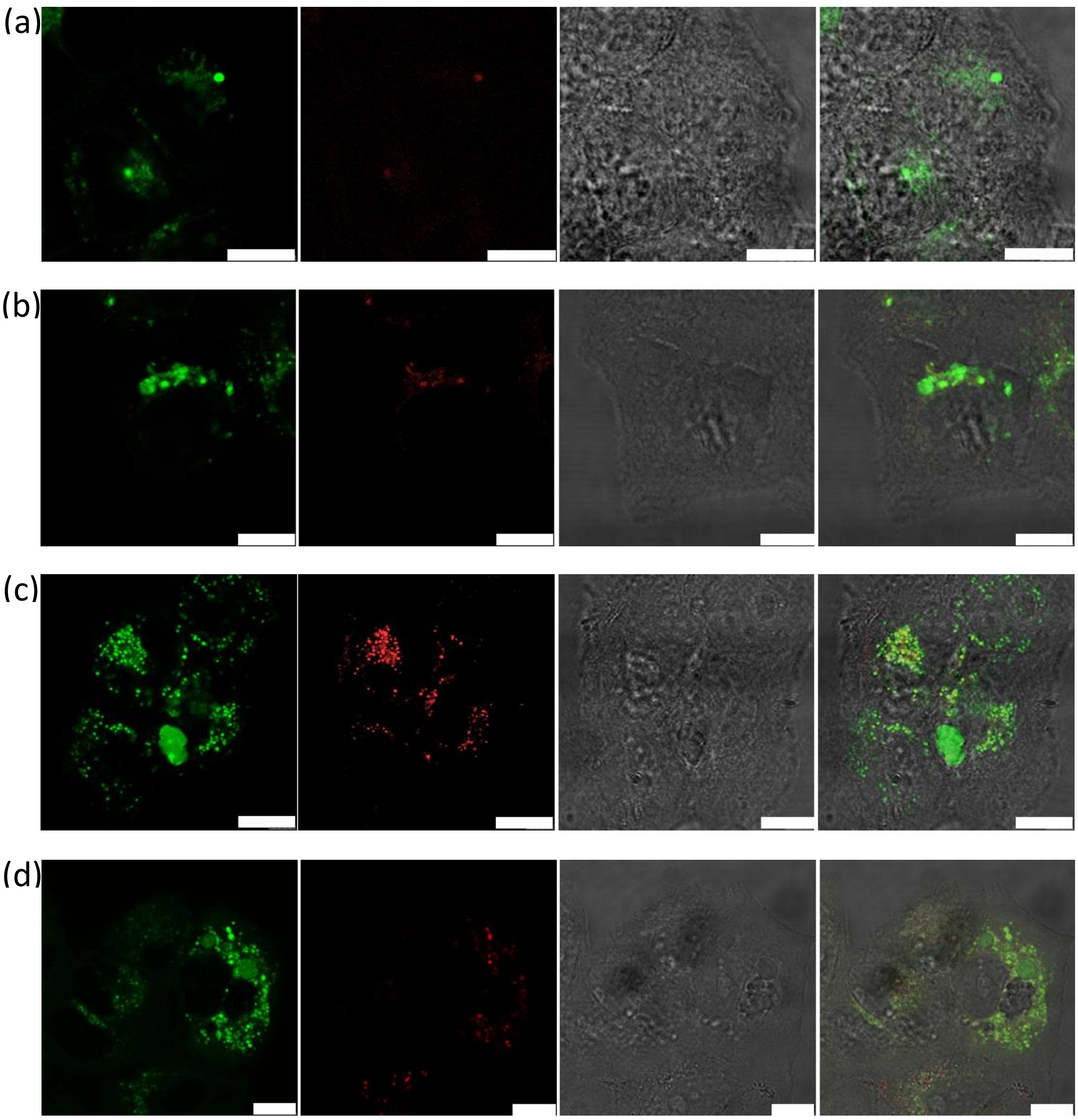

Figure S8. Confocal images of HeLa cells fed with needle-shaped NPs at different concentration for 24 hours. (a) 25, (b) 50, (c) 100, (d) 250 μg/mL. (Images from left to right: lysosome in green, NPs in red, transmission and overlaid. Scale bar: 10 μm).

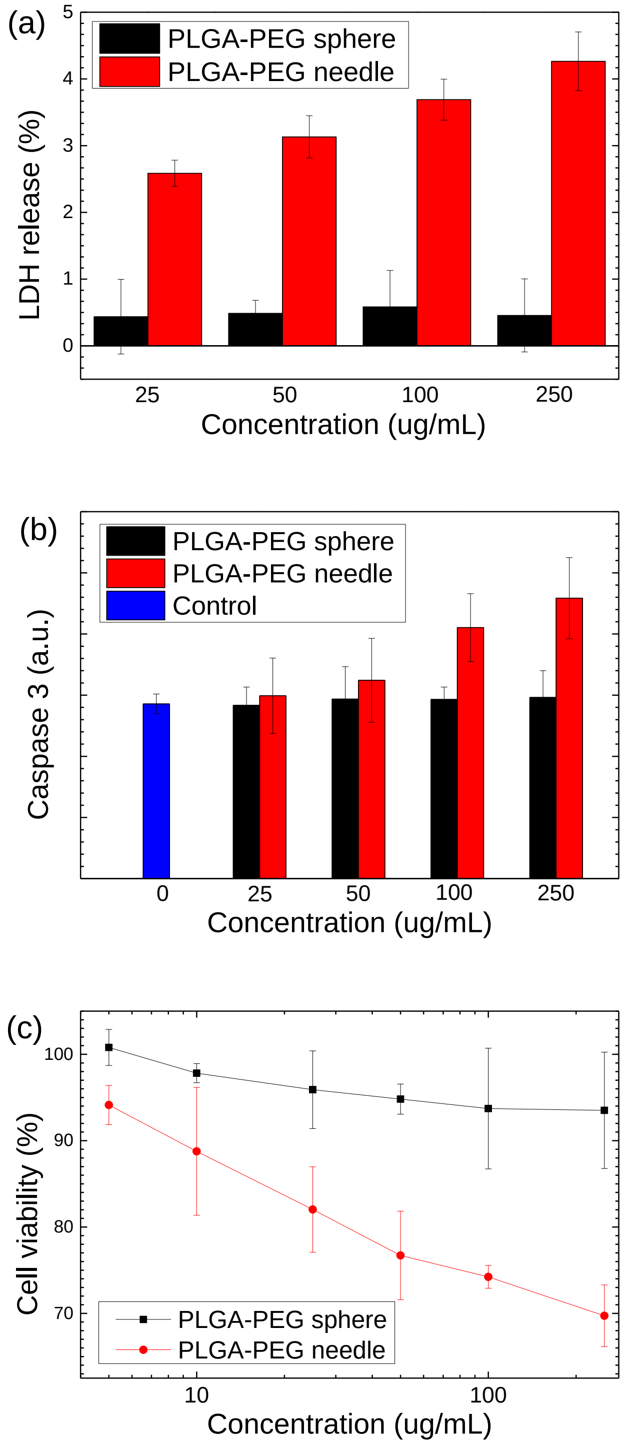

Figure S9. Shape dependent PLGA NPs cytotoxicity of HeLa cells fed with spherical- and needle-shaped NPs. (a) LDH release (normalized to lysed control cell), (b) Caspase 3 activity and (c) MTT assay after HeLa cells were fed with spherical- and needle-shaped PLGA-PEG NPs for 24 hours.

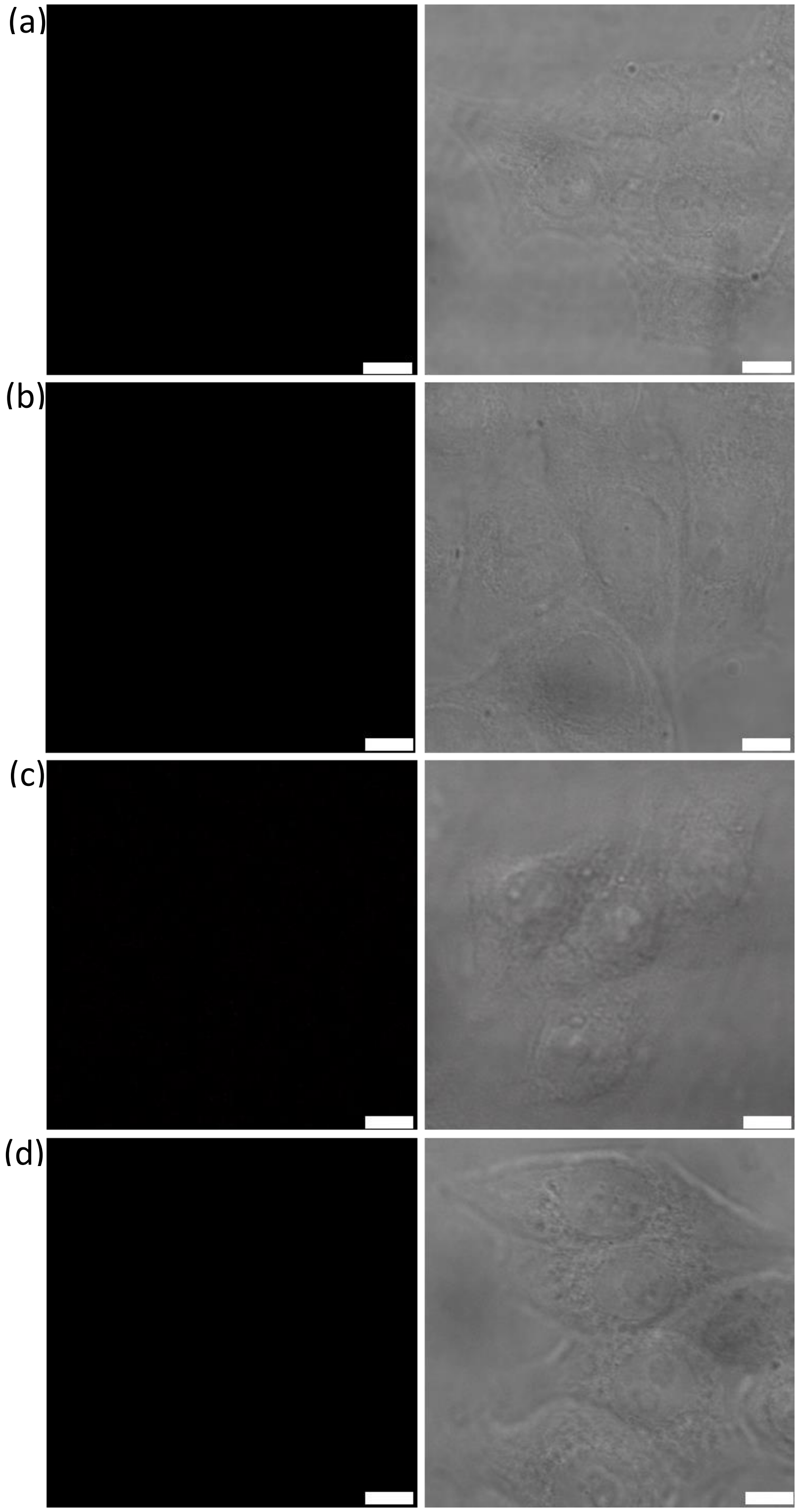

Figure S10. Confocal images of HepG2 cells (stained with TUNEL Red) after their being fed with spherical-shaped NPs at (a) 25, (b) 50, (c) 100 and (d) 250 μg/mL for 24 hours. (Red: TUNEL. Scale bar: 10 μm).

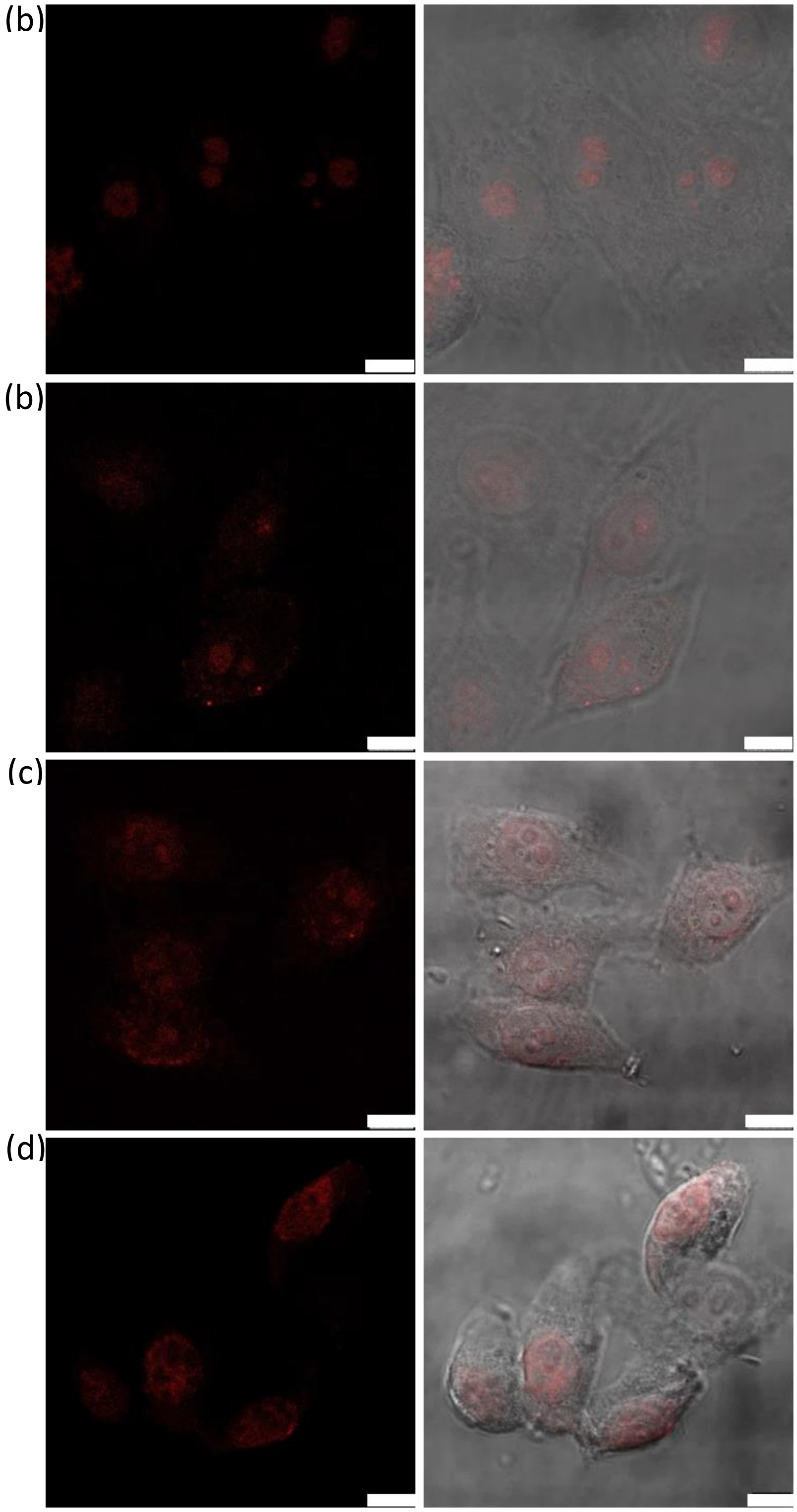

Figure S11. Confocal images of HepG2 cells (stained with TUNEL Red) after their being fed with needle-shaped NPs at (a) 25, (b) 50, (c) 100 and (d) 250 μg/mL for 24 hours. (Red: TUNEL. Scale bar: 10 μm).

1. Cheng, J. *et al.* Formulation of functionalized PLGA–PEG nanoparticles for in vivo targeted drug delivery. *Biomaterials* **28,** 869–876 (2007).
2. Xu, P. *et al.* Intracellular Drug Delivery by Poly(lactic- *co* -glycolic acid) Nanoparticles, Revisited. *Mol. Pharm.* **6,** 190–201 (2009).
3. Champion, J. A. & Mitragotri, S. Role of target geometry in phagocytosis. *Proc. Natl. Acad. Sci. U. S. A.* **103,** 4930–4934 (2006).
